# Self-Monitoring App Preferences for Sun Protection: Discrete Choice Experiment Survey Analysis

**DOI:** 10.2196/18889

**Published:** 2020-11-27

**Authors:** Vasileios Nittas, Margot Mütsch, Julia Braun, Milo Alan Puhan

**Affiliations:** 1 Epidemiology, Biostatistics, and Prevention Institute University of Zurich Zurich Switzerland

**Keywords:** preventive medicine, mHealth, telemedicine, health informatics, health economics, preferences, sun protection

## Abstract

**Background:**

The availability and use of health apps continues to increase, revolutionizing the way mobile health interventions are delivered. Apps are increasingly used to prevent disease, improve well-being, and promote healthy behavior. On a similar rise is the incidence of skin cancers. Much of the underlying risk can be prevented through behavior change and adequate sun protection. Self-monitoring apps have the potential to facilitate prevention by measuring risk (eg, sun intensity) and encouraging protective behavior (eg, seeking shade).

**Objective:**

Our aim was to assess health care consumer preferences for sun protection with a self-monitoring app that tracks the duration and intensity of sun exposure and provides feedback on when and how to protect the skin.

**Methods:**

We conducted an unlabeled discrete choice experiment with 8 unique choice tasks, in which participants chose among 2 app alternatives, consisting of 5 preidentified 2-level attributes (self-monitoring method, privacy control, data sharing with health care provides, reminder customizability, and costs) that were the result of a multistep and multistakeholder qualitative approach. Participant preferences, and thus, the relative importance of attributes and their levels were estimated using conditional logit modeling. Analyses consisted of 200 usable surveys, yielding 3196 observations.

**Results:**

Our respondents strongly preferred automatic over manually operated self-monitoring (odds ratio [OR] 2.37, 95% CI 2.06-2.72) and no cost over a single payment of 3 Swiss francs (OR 1.72, 95% CI 1.49-1.99). They also preferred having over not having the option of sharing their data with a health care provider of their choice (OR 1.66, 95% CI 1.40-1.97), repeated over single user consents, whenever app data are shared with commercial thirds (OR 1.57, 95% CI 1.31-1.88), and customizable over noncustomizable reminders (OR 1.30, 95% CI 1.09-1.54). While most participants favored thorough privacy infrastructures, the attribute of privacy control was a relatively weak driver of app choice. The attribute of self-monitoring method significantly interacted with gender and perceived personal usefulness of health apps, suggesting that female gender and lower perceived usefulness are associated with relatively weaker preferences for automatic self-monitoring.

**Conclusions:**

Based on the preferences of our respondents, we found that the utility of a self-monitoring sun protection app can be increased if the app is simple and adjustable; requires minimal effort, time, or expense; and has an interoperable design and thorough privacy infrastructure. Similar features might be desirable for preventive health apps in other areas, paving the way for future discrete choice experiments. Nonetheless, to fully understand these preference dynamics, further qualitative or mixed method research on mobile self-monitoring-based sun protection and broader preventive mobile self-monitoring is required.

**International Registered Report Identifier (IRRID):**

RR2-10.2196/16087

## Introduction

### Background

The global increase in smartphone ownership is unprecedented [1]. Worldwide, approximately seven billion people are estimated to own a mobile phone, more than half of which are smartphones [1,2]. Simultaneously, increased access to the internet and rising advocacy for person-centered care promote a rapidly growing mobile health (mHealth) market [3]. Inevitably, the availability of free or low-cost health apps has increased, surpassing 300,000 as of 2017 [3,4]. Shifting beyond mere SMS, the multifunctionality of apps has the potential to revolutionize the way mHealth interventions are delivered [5]. They can be used to prevent disease and facilitate well-being, such as monitoring behavior and promoting healthy life-styles; to manage existing conditions, such as providing behavioral therapies and assisting medication adherence; and to support rehabilitation and health care access [5-7].

### Mobile Self-Monitoring Apps for Sun Protection

Skin diseases, such as skin cancers, that are caused by sun exposure are also on the rise [8]. Primarily prevalent in Caucasian, fair-skinned populations, melanoma and nonmelanoma skin cancers increasingly affect younger age groups [8]. Beyond uncontrollable risk factors such as genetics, the most significant and avoidable risk factor is exposure to ultraviolet light (eg, sun, tanning beds) [8]. Targeted behavior changes, such as improved sun protection (eg, shade, protective clothing, sunscreen use during exposure to the sun) as well as full avoidance of tanning beds, are key elements to the mitigation of a growing public health burden [8].

Primary prevention (eg, wellness, physical activity, diet, healthy behaviors) accounts for a large proportion of health apps [5]. Most of these have functions to collect and analyze health-related data, enabling simple and continuous self-monitoring [5]. These trends lead to an ever-growing volume of electronic patient-generated health data, defined as nonclinical health information [9]. Using mHealth apps to self-monitor is expected to facilitate prevention by measuring risk exposure (eg, sun intensity), which in turn can increase risk awareness and encourage healthy behavior (eg, seeking shade, applying sunscreen) [5,10]. Acknowledging the reach and potential of self-monitoring apps and the increasing burden of preventable skin cancers, efforts to understand how the former are to be designed and utilized to successfully prevent the latter are timelier than ever before.

### Consumer Preferences for Sun Protection With Self-Monitoring Apps

Nonetheless, health apps are often deleted or abandoned rapidly as they fail to meet user needs and expectations [11]. One approach to meeting these is by exploring the preferences of prospective users. This is particularly important for health apps that tend to have a niche and often periodic use, such as in the case of sun protection apps. Within often limited time windows, such apps need to deliver a certain value in order to be considered for download and adequate use. In addition, both self-monitoring as well as disease prevention predominantly depend on individual motivation (acceptance and engagement), which is in turn linked to individual preferences [12-14]. Despite that, consumer preferences for self-monitoring-based sun protection apps have not yet been adequately explored. We aim to fill this gap by reporting the findings on the preferences of 200 health care consumers.

### Aims

In the context of sun protection, this study aimed to assess which attributes of a theoretical self-monitoring app are perceived as more or less important by health care consumers. This was guided by the following objectives: (1) to elicit how consumer preferences are distributed among a set of preidentified app attributes and (2) to assess whether preferences vary across individual characteristics, such as age, gender, education, perceived health and perceived health app usefulness.

## Methods

### Study Design

Consumer preferences were elicited with a self-administered discrete choice experiment survey. Discrete choice experiments provide respondents with 2 or more alternative options of a product or service (in our case, 2 alternatives of the same app), asking them to repeatedly choose the most desired one [15,16]. All alternatives are defined by the same attributes, however, contain different manifestations (levels) of these. Every attribute included in a discrete choice experiment takes 2 or more forms. For example, if cost is included as an attribute, its levels could take 2 (or more) forms, such as free or 3 Swiss francs. These repeated choices allow for statistically eliciting which of those attributes (and their levels) are perceived as more valuable, and thus, yield the highest utility [16]. A person’s perceived utility of an attribute depends on the attribute’s level (eg, for the cost attribute, a less expensive app might be perceived as more valuable than a more expensive app). While traditionally rooted in economic research, discrete choice experiments are gradually gaining popularity in digital health research [15]. Our methodology follows the guidelines reported by the ISPOR Good Research Practices for Conjoint Analysis Task Force [17]. Our study design followed these 5 steps: (1) qualitative work for the identification of key attributes, (2) survey and scenario development, (3) piloting and survey adjustments, (4) survey administration and data collection, and (5) choice data analysis. A detailed description of the applied methodology, as well as a justification of our methodological rationale have been reported in a previously published registered protocol [18].

### Attribute Identification

**Figure 1:**
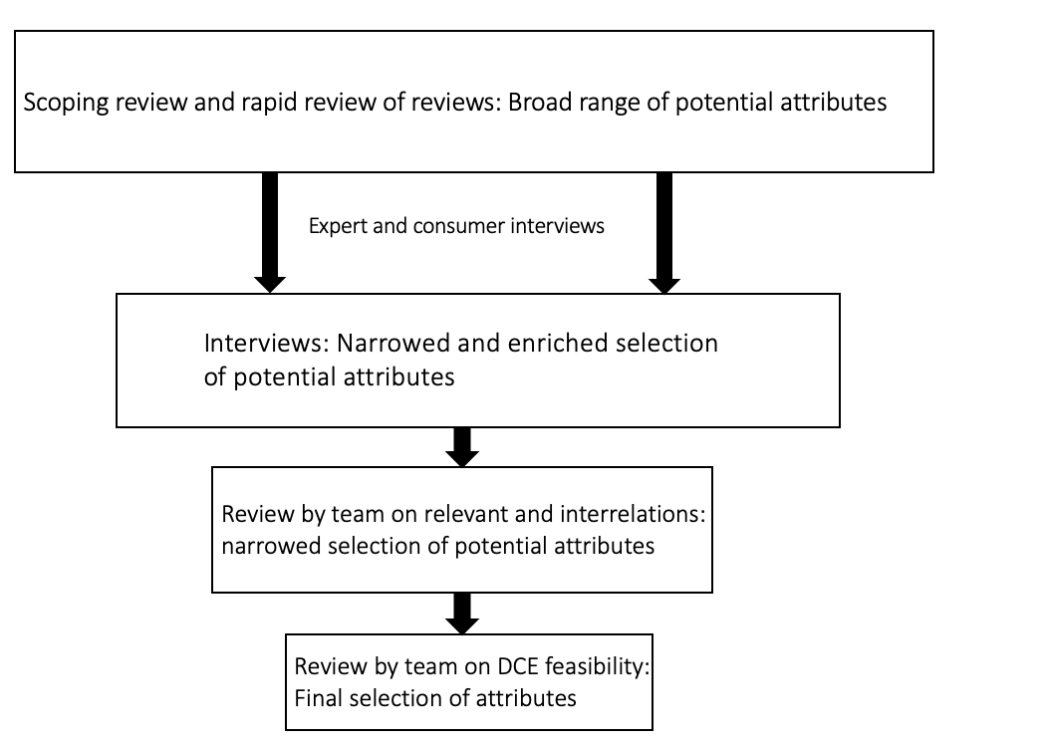
Attribute selection process. DCE: discrete choice experiment.

We followed good practice standards and identified the attributes of our discrete choice experiment by (1) using the results of the previously conducted systematic scoping review, (2) conducting a rapid review of systematic reviews on the use of self-monitoring data for primary prevention [19], (3) completing 13 semistructured expert interviews and 12 health care consumer interviews as well as (4) 2 internal review rounds (Figure 1) [18].

We selected experts that covered all relevant expertise fields, including digital self-monitoring, digital prevention, eHealth ethics, clinical science, and citizen science [18]. Health care consumer interviews were gender balanced and consisted of adults. Interviews were recorded, transcribed, and analyzed following a mixed deductive and inductive approach. The literature reviews led to a broad range of potential attributes, which was narrowed down and complemented by the interviews. This was followed by 2 internal review rounds, assessing the relevance, interrelations, and feasibility of identified attributes, leading to the selection and inclusion of the following 5 attributes: (1) method of self-monitoring, (2) privacy control, (3) data sharing with a health care provider, (4) reminder customizability, and (5) costs. An overview of included attributes with their levels and definitions is provided in Table 1.

For each attribute, we chose 2 realistic, relatable, and easy-to-understand levels, primarily based on our literature and interview findings. Attributes and levels were purposively kept simple and unambiguous, ensuring that all respondents could complete the survey with minimum effort. Long or too complex discrete choice experiments can pose a disproportionate cognitive burden on respondents, which increases the risk of incomplete or inaccurate responses. We hypothesize that health care consumers will prefer automatic over manual self-monitoring, customizable over noncustomizable reminders, and no cost over a single payment have all been confirmed. For the other two attributes, a hypothesis cannot be clearly formulated.

**Table 1:** Identified attributes and attribute levels.

Attributes	Descriptions and attribute levels	
**Self-monitoring method**	**How would you like your data to be collected?**	
		No manual entry (automatic entry)
		Manual entry once a day
**Privacy control**	**If your data are being shared with third commercial parties, how would you like to control when and with whom your data are shared?**	
		I will only receive information on potential receivers once and will be asked to provide informed consent once
		I will be informed and provide consent whenever my data are provided to third parties
**Data sharing with health care provider**	**Would you like to share the data collected with your health care provider, to be discussed at your next visit?**	
		Yes
		No
**Reminder customizability**	**How would you prefer the times and frequency of your reminders to be set?**	
		I set the time and frequency of my reminders myself (customizable)
		The app sets the times and frequency of reminders automatically, based on my data
**Costs**	**Are there any costs associated with downloading the app, and if yes, how high are these?**	
		Free
		One-time 3 Swiss francs

### Survey Design and Scenario

**Figure 2:**
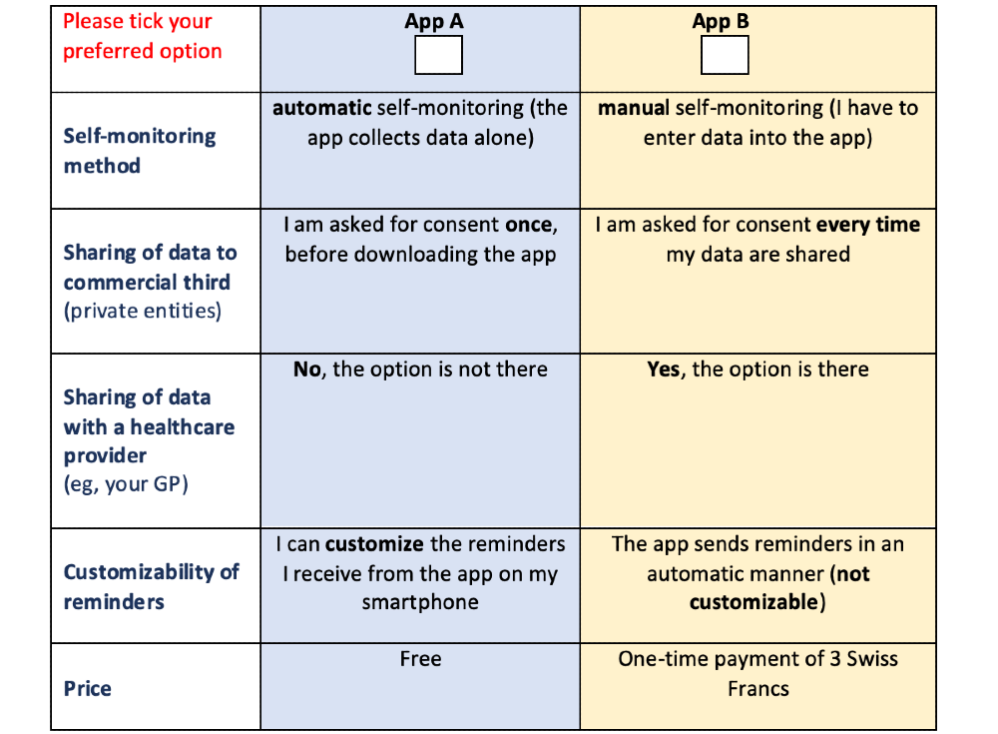
Choice set.

Our 5 attributes, each with 2 levels, would lead to a full factorial design of 32 (2^5^ = 32) possible choice sets [20]. However, asking participants to make 32 discrete choices would be highly impractical, due to limited time and high complexity. Hence, we followed common practice and reduced the survey to a subset of possible choice sets, known as fractional factorial design. Based on D-optimality criteria, the resulting fractional factorial design consisted of 8 choice sets, each providing 2 app alternatives [21,22]. In addition to the 8 fractional factorial design choice sets, we decided to include a ninth set, which was used to assess the quality of responses. The ninth choice set was identical to a previous one, allowing us to assess whether responses were consistent (same app chosen for both identical sets) or not (different app chosen between the 2 identical sets). An example choice set is provided in Figure 2.

Framing a discrete choice experiment around a well-defined hypothetical scenario helps participants understand the survey and improves the accuracy of their choices. After being introduced to the topic, each participant was asked to imagine that summer was ahead and a preventive app that supports sun protection, helps with skin cancer prevention and promotes overall skin health was being recommended. The app would monitor daily duration and intensity of sun exposure and provide targeted feedback on when and how to protect. The 2 app options were provided in an unlabeled manner (as App 1 and App 2) in order to entirely steer the focus of our participants on the attributes and their levels [23].

The discrete choice experiment was generated in German and proofread by a native speaker. The reviewer was asked to pay attention on word choice, grammar, and punctuation as well as overall understanding.

### Piloting

We piloted the initial discrete choice experiment face to face with 8 participants (5 males and 3 females), recruited in the waiting room of the University of Zurich Travel Clinic. Participants received the full questionnaire and were asked to share their thoughts while completing it. Through this think-aloud process, we captured how the discrete choice experiment’s wording and layout could be improved to facilitate its feasibility. All conversations were recorded. Time to completion was measured to ensure that the survey could be answered within a 20-minute time frame. The limit was set at 20 minutes to be below the average waiting time in the travel clinic’s waiting room, where final recruitment occurred. Participants were also asked to provide feedback on the survey’s relevance and ease of answering. Prior to that, pilot participants provided written informed consent and received written as well as verbal information on the study’s aims and methodology. All participants considered the discrete choice experiment as interesting and relevant. Most commented on unclear terminology and expressed confusion of how the survey has to be answered. This led to (1) adjustments in the survey’s wording, by choosing simple and self-explanatory terms, (2) changes in the survey’s layout to reduce confusions of how choices should be made (eg, using colors to differentiate the 2 app options), and (3) revisions of the survey’s instructions to ensure that participants understand how the survey has to be completed.

### Survey Administration

The survey was administered at the University of Zurich Travel Clinic. On arrival at the clinic’s waiting room, participants were informed about the option to participate, both verbally and through a flyer. Interested and eligible participants were asked to fill out the paper-based discrete choice experiment in the waiting room, where a member of the team was present to clarify questions. Eligibility required a minimum of 18 years of age, no current chronic conditions, a good understanding of German, and smartphone ownership. As scientific consensus on sample size estimation of discrete choice experiments is lacking, we used a commonly applied rule of thumb, proposed by Johnson and Orme [24]. Thus, based on the number of choice tasks, alternatives, and analysis cells, we set our sample size at 200 participants. The paper-based discrete choice experiment’s first pages aimed to inform participants, providing concise and easy-to-understand information on the study’s aims, the topic of sun protection, each of the included attributes (with pictographs), and how to complete the discrete choice experiment.

Upon completion, participants were thanked for their time and effort with a sunscreen product. We also collected participant characteristics, including gender, age, highest attained education, perceived health, and perceived health app usefulness. We did not collect any personal identification or sensitive health information. Eligibility had to be confirmed by ticking the box “I confirm that I fulfill all eligibility criteria listed above” before answering the survey.

### Analysis of Choice Data

Individual characteristics were explored descriptively. Participant preferences were estimated using conditional logit modeling, as proposed by McFadden [25]. The conditional logit regression is in line with random utility theory, according to which the utility a respondent *n*=1,2,…,200 derives from an alternative *j* consist of a systematic (representative) and a random component. The systematic component is denoted by 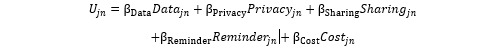


where β_Data_, β_Privacy_, β_Sharing_, β_Reminder_, and β_Cost_ are the unknown parameters associated with the 5 attribute variables. *Data_jn_* takes the value 0 or 1 corresponding to manual entry or automatic entry, respectively; *Privacy_jn_* takes the value of 0 or 1 if consent for sharing data with commercial third parties is only given once or if consent is given on every sharing occasion, respectively; *Sharing_jn_* takes the value of 0 or 1 if data are not shared with a chosen health care provider or data are shared with a chosen health care provider, respectively; *Reminder_jn_* takes the value of 0 or 1 if noncustomizable or manually customizable, respectively; and *Cost_jn_* takes the value of 0 or 1 if the app has a one-time cost of 3 Swiss francs or if the app is free, respectively. The results are reported as odds ratios (ORs), indicating relative importance of attribute levels compared to reference categories and their associated 95% confidence intervals. Although our survey entailed a binary cost option (free, 3 Swiss francs), we additionally ran the model with cost treated as a continuous variable, allowing it to express shifts in preference weights per 1 Swiss Franc change.

As a person’s characteristics may influence their choice, we decided to individually test all collected individual characteristics for interactions with our attributes. These were identified and tested by repeatedly adding single interaction terms into the model. Multiple comparisons were counteracted through Bonferroni corrections (α=.002). Significant interaction terms are reported individually. A mixed multinomial regression model was performed to explore potential preference heterogeneity. Here, we only assumed the effect of price to be random, as it is the only attribute where the assumption of an underlying latent continuous distribution is possible.

We additionally checked whether the 2 identical choice sets were answered consistently. In line with the axiom of completeness, participants who provide consistent answers are expected to choose the same alternative twice [17,26]. As directly excluding participants is not always recommended, we calculated percentages of inconsistent responses, assessed their distribution across individual participant characteristics and performed a sensitivity analysis comparing the results of our analyses with and without inconsistent responses [17,23,27]. Due to the small number of missing observations (4/3200, 0.13%) we decided to not perform multiple imputations. All analyses were performed in R (version 3.6.1).

## Results

### Participant Characteristics

Participant characteristic are presented in Table 2. Our sample (N=200) was unintentionally gender-balanced and yielded 3196 observations. Most participants were between 18 and 39 years old (135/200, 67.5%); had a tertiary education level (141/200, 70.5%), such as university and professional education degrees; rated their overall health as quite good (107/200, 53.5%) or very good (88/200, 44%); and had a positive perception (106/200, 53%) of apps’ usefulness to their personal health.

**Table 2:** Participant characteristics.

Variable		Participants (N=200), n (%)
**Gender**		
	Male	100 (50.0)
	Female	100 (50.0)
**Age (years)**		
	18-39	135 (67.5)
	40-60	53 (26.5)
	61-79	11(5.5)
	80+	1 (0.5)
**Highest education level**		
	Primary	5 (2.5)
	Secondary	51 (25.5)
	Tertiary	141 (70.5)
	Other	3 (1.5)
**Perceived health status (How would you rate your current overall health?)**		
	Very good	88 (44.0)
	Quite good	107 (53.5)
	Average	5 (2.5)
	Quite bad	0 (0)
	Bad	0 (0)
**Perceived health app usefulness (Do you agree that apps could help you promote your health?)**		
	Fully agree	24 (12.0)
	Agree	106 (53.0)
	Uncertain	56 (28.0)
	Disagree	10 (5.0)
	Totally disagree	4 (2.0)

### Main Effects

**Figure 3:**
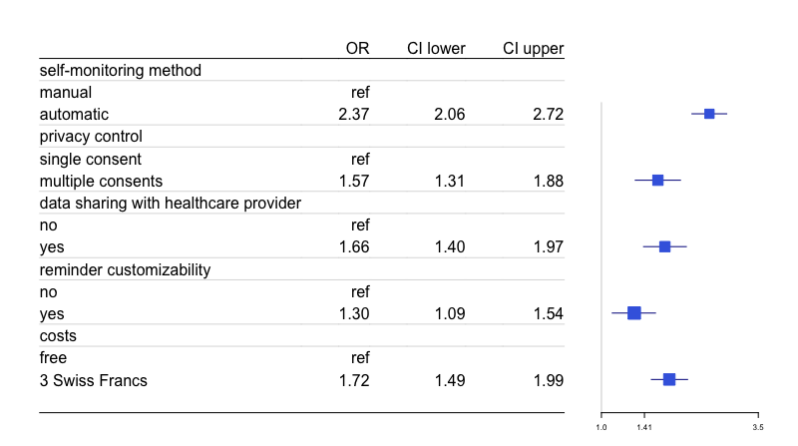
Forest plot from conditional logit regression (N=200). OR: odds ratio.

The results from the conditional logit model without interaction effects (model 1), are presented in Table 3 and in Figure 3. Overall, all 5 attributes contributed to consumer choices and overall preferences. The strongest driver of choice—denoted by the largest value of the OR—was the app’s self-monitoring method, followed by cost, the option of data sharing with a health care provider, privacy control function, and reminder customizability (the least strong driver).

**Table 3:** Discrete choice experiment results from conditional logit regression (N=200).

Attribute and levels		OR^a^ (95% CI)	*P* value
**Self-monitoring method**			<.001
	Manual	Reference	
	Automatic	2.37 (2.06-2.72)	
**Privacy control**			<.001
	Single consent	Reference	
	Multiple consents	1.57 (1.31-1.88)	
**Data sharing with health care provider**			<.001
	No	Reference	
	Yes	1.66 (1.40-1.97)	
**Reminder customizability**			<.01
	No	Reference	
	Yes	1.30 (1.09-1.54)	
**Costs**			<.001
	Free	Reference	
	3 Swiss francs	1.72 (1.49-1.99)	

^a^OR: odds ratio.

Our respondents strongly preferred automatic self-monitoring, with higher odds of choosing an app if it collected data automatically than if it required manual data entry (OR 2.37, 95% CI 2.06-2.72). Similarly, our respondents preferred no cost, with higher odds of choosing a free app than one requiring a one-time payment of 3 Swiss francs (OR 1.72, 95% CI 1.49-1.99). Modeling price as a continuous variable indicated that the odds of choosing an app alternative were reduced by 17% (OR 0.83, 95% CI 0.79-0.88) per 1 Swiss Franc increase in app cost. Respondents also preferred having the option of sharing their health data with a chosen health care provider than not having that sharing option (OR 1.66, 95% CI 1.40-1.97). Similarly favored was a thorough privacy control, with multiple user consents (given whenever app data are shared with third commercial parties) increasing the odds of choosing an alternative relative to one in which only a single consent was required (eg, after downloading the app) (OR 1.57, 95% CI 1.31-1.88). Finally, the setting of sun protection reminders was the weakest, yet still a driver of choice, with customizable reminders being preferred over automatic reminders increasing the odds of choosing an app (OR 1.30, 95% CI 1.09-1.54). These findings were comparable to those of the mixed multinomial model (model 2), considering that as well as the unlabeled design and binary choice nature of our discrete choice experiment, we decided to limit reporting to the conditional logit’s output and retain the mixed multinomial model as a sensitivity analysis.

### Interaction Effects

After Bonferroni correction, only gender and perceived health app usefulness showed an interaction, in both cases with the self-monitoring method attribute. While both men and women preferred an app that generates their health data automatically, women had only half the odds of choosing an app alternative that offers that option (men: OR 3.42, 95% CI 2.78-4.21; women: OR 1.71, 95% CI 1.42-2.05). Those who were uncertain about whether apps can be helpful in promoting their health (neutral attitude) had less than half the odds of choosing an automatic self-monitoring app compared to those who fully agreed and thus had a positive perception of health app usefulness. Nonetheless, both groups preferred automatic generation over manual (full agreement: OR 4.19, 95% CI 2.71-6.48; neutral: OR 1.83, 95% CI 1.38-2.42). However, this effect changed direction for the group that fully disagreed with the statement that apps can be useful for personal health promotion (OR 0.32, 95% CI 0.11-0.92).

### Inconsistent Responses

Eleven participants (11/200, 5.5%) provided inconsistent responses, that is, they answered the 2 identical questions differently; 6 were female and 5 were male. Most inconsistent responses were provided by participants between the age of 19 and 39 years (n=8), with a tertiary education (n=9), and who either had a rather positive or neutral perception (n=11) of apps’ usefulness to their personal health. Comparing the results from the conditional logit model with and without the eleven inconsistent responders yielded comparable results; therefore, we decided not to exclude any participants.

## Discussion

### Principal Findings

This discrete choice experiment explored the preferences of healthy consumers for sun protection with a self-monitoring app. The survey included 5 attributes, presented as 2 unlabeled app alternatives (App A and App B) across 8 choice tasks (and 1 additional repeated task). We found that all 5 attributes influenced the choices of our participants. That confirms our preparatory qualitative work and the importance of these attributes for self-monitoring-based sun protection apps. Our hypotheses that health care consumers would prefer automatic over manual self-monitoring, customizable over noncustomizable reminders, and no cost over a single payment have all been confirmed. The method of self-monitoring had the strongest influence on app choice, followed by cost, data sharing with a health care provider, privacy control, and the customizability of reminders. Secondary analyses suggested that the strength of preference for automatic self-monitoring was influenced by gender and perceived usefulness of health apps.

### Effort and Ease of Use

Being well-aligned with current evidence, our participants mostly preferred sun protection apps that monitored health data automatically. Manual data collection requires time and effort, which is often perceived as tedious and inconvenient, reducing simplicity, ease-of-use and motivation [28,29]. Furthermore, the use of a sun protection app is more likely to occur during leisure time (eg, outdoor activities, holidays), which naturally makes manual monitoring a less attractive option. The underlying concept of effort and simplicity is recurring in the literature. For example, during the development of SunSmart, an ultraviolet monitoring and risk communication app, as well as mISkin, a sun protection app for those going on holidays, participants highly valued ease-of-use, which was described by the authors as a key feature [30,31]. This also holds for other prevention areas that require frequent and often complex monitoring, such as dietary intake and weight loss [32]. Our findings are potentially reinforced by the fact that self-monitoring sensors have become more accurate over the years, which in turn increases trust in automatic self-monitoring and facilitates trends toward easy-to-carry, and in fact, hidden monitoring devices [33,34].

### Data Sharing

Although privacy concerns are often listed as key barriers of mobile self-monitoring apps, the attribute of privacy control, in the form of providing consents for data sharing with commercial parties, was relatively weak in influencing the app choices of our respondents [28]. Again, this may be explained (1) by the nature of sun protection behavior data and the good health status of our sample and (2) by the effort and time that is associated with the provision of multiple consents. Another factor that makes multiple consents less attractive is the information overload with which they are often associated [35]. Although the attribute did not drive choice as strongly as cost or the method of self-monitoring, our sample preferred providing multiple consents, repeated whenever their data are shared with commercial third parties. Our second data sharing attribute targeted the information flow between consumer and health care provider (eg, general practitioner, dermatologist). Having that sharing option clearly influenced app choice. On average, being able to share data with a doctor was more important than the app’s privacy functions. This is a surprising and very interesting finding if we consider that our respondents were healthy and that such sharing functions are not yet widely available [28]. However, it potentially underlines the value and trust that app consumers place in interacting with health care professionals. Anderson et al [36] explored user experiences with self-care targeted mHealth apps and identified a similar pattern. Many users would have preferred the option of sharing their data with health care providers, allowing for a better-informed care and reduced visits [36]. In the context of sun protection, this preference might also be reinforced by the very nature of sun protection behavior data, which are not particularly controversial, stigmatized, or sensitive.

### Reminders: Motivation Versus Nuisance

Equally unexpected was the relative importance of reminder customization, which was the weakest among the 5 attributes in influencing app choice. Reminders are a widely used mobile self-monitoring feature, highly applicable across all health areas. The insensitivity of reminders (eg, too frequent) has been recurrently cited as a barrier of mHealth use [28]. On average, our respondents preferred to be able to customize their app reminders, which may suggest previous experience with too many (or too few) reminders. The efficacy of reminders in health behavior change has been repeatedly demonstrated and so has their potential to be a nuisance factor and achieve the opposite [37-39]. Customizability is, therefore, often referred to as a key element of effective reminder systems. For example, during the iterative development of the mISkin sun protection app, prospective users expressed their preference to be able to personalize the frequency of reminder messages, as the default failed to meet their needs [31]. Beyond sun protection, a recently evaluated smoking cessation app identified that nonpersonalized reminders were primarily perceived as an irritation [37]. Similarly, reviews in the context of medication adherence and vaccinations described reminders, as well as reminder customizability, as being of key importance to user preferences and effectiveness [38,39].

### Free First

On average, our respondents preferred free apps, which is an expected finding for 2 main reasons: First, healthy consumers are likely to use a sun protection app only periodically, such as during summer or sunny holidays. That potentially decreases an app’s perceived value and the price someone is willing to pay for using it [40]. Second, our sample was predominantly young, which again might have influenced the overall willingness to pay. Gowin and colleagues [41] formatively explored the use of preventive and health promoting apps among college students, identifying that half of them would not be willing to pay, even if the amount was very small. This might be because of the high number of free apps, as well as because of different expectations by younger age groups, formed by their very early technology exposure [41]. Finally, younger age is associated with lower income, and thus, lower motivation to pay [42].

### Limitations

Our findings should be considered with regard to the following limitations: First, recruitment occurred at a single center (Travel Clinic of the University of Zurich) and was subject to predefined inclusion criteria, for which reason our findings may not be generalizable. Second, despite the methodological robustness of discrete choice experiments, they maintain their feasibility by focusing on a limited number of attributes. Inherently, this reduces the discrete choice experiment’s capacity to fully capture all possible influences of choice, whether individual or broader. We counteracted that by following a thorough qualitative attribute identification process and selecting attributes that are modifiable, are relevant, and cover an important array of mobile self-monitoring characteristics. Nonetheless, to fully understand these, we call for follow-up qualitative or mixed method research on mobile self-monitored sun protection and broad preventive mobile self-monitoring across population samples.

### Conclusions

All 5 discrete choice experiment attributes influenced the choices of our respondents. Our sample’s strongest preference was for sun protection apps that enabled automatic self-monitoring, which seemed to be even stronger for male respondents as well as those with an overall positive perception on the usefulness of health apps. Apps that were free of cost were the second most preferred choices. Furthermore, on average, an app was more likely to be chosen if it allowed for the in-app sharing of self-monitoring data with a chosen health care provider, for repeated user consents whenever data are shared with commercial third parties, and for reminders that can be customized. Surprisingly, the future-oriented function of data sharing with a health care provider was more important than the app’s privacy control or reminder customizability. This underlines the potential value of connecting apps with trusted health care providers. Based on these findings, a preference- and user-sensitive self-monitoring app for sun protection should be simple and adjustable; require minimal effort, time or expenses; be interoperable; and have thorough as well as transparent privacy infrastructure. Factors that might partially explain our findings are our sample’s good health and the rather periodic use of sun protection apps (eg, during summer). Similar features might be desirable for preventive health apps in other areas, paving the way for future discrete choice experiments.

## Abbreviations

CI: confidence interval
mHealth: mobile health
OR: odds ratio

## References

[1] Taylor K, Silver L (2019). Smartphone Ownership is Growing Rapidly Around the World, but Not Always Equally. Pew Research Center.

[2] (2015). Measuring the Information Society Report 2015. International Telecommunication Union.

[3] Poushter J (2016). Smartphone Ownership and Internet Usage Continues to Climb in Emerging Economies But advanced economies still have higher rates of technology use. Pew Research Center.

[4] (2017). mHealth Economics 2017 – Current Status and Future Trends in Mobile Health. Research2Guidance.

[5] Kao CK, Liebovitz DM (2017). Consumer Mobile Health Apps: Current State, Barriers, and Future Directions. PM R.

[6] Zhou L, Bao J, Watzlaf V, Parmanto B (2019). Barriers to and Facilitators of the Use of Mobile Health Apps From a Security Perspective: Mixed-Methods Study. JMIR Mhealth Uhealth.

[7] Roess A (2017). The Promise, Growth, and Reality of Mobile Health - Another Data-free Zone. N Engl J Med.

[8] About Melanoma. Melanoma Research Alliance.

[9] Shapiro M, Johnston D, Wald J, Mon D (2012). Patient-Generated Health Data - White Paper. RTI International.

[10] Abril EP (2016). Tracking Myself: Assessing the Contribution of Mobile Technologies for Self-Trackers of Weight, Diet, or Exercise. J Health Commun.

[11] Murnane EL, Huffaker D, Kossinets G (2015). Mobile health apps: Adoption, adherence, and abandonment.

[12] Munson SA, Consolvo S (2012). Exploring Goal-setting, Rewards, Self-monitoring,Sharing to Motivate Physical Activity.

[13] Buchwald A, Letner A, Urbach N, Von Entreß-Fürsteneck M (2018). Insights into personal ICT use: Understanding continuance and discontinuance of wearable self-tracking devices.

[14] Short CE, DeSmet A, Woods C, Williams SL, Maher C, Middelweerd A, Müller AM, Wark PA, Vandelanotte C, Poppe L, Hingle MD, Crutzen R (2018). Measuring Engagement in eHealth and mHealth Behavior Change Interventions: Viewpoint of Methodologies. J Med Internet Res.

[15] Ryan M, Farrar S (2000). Using conjoint analysis to elicit preferences for health care. BMJ.

[16] Ryan M, Bate A, Eastmond CJ, Ludbrook A (2001). Use of discrete choice experiments to elicit preferences. Qual Health Care.

[17] Bridges JFP, Hauber AB, Marshall D, Lloyd A, Prosser LA, Regier DA, Johnson FR, Mauskopf J (2011). Conjoint analysis applications in health--a checklist: a report of the ISPOR Good Research Practices for Conjoint Analysis Task Force. Value Health.

[18] Nittas V, Mütsch M, Puhan MA (2020). Preferences for Sun Protection With a Self-Monitoring App: Protocol of a Discrete Choice Experiment Study. JMIR Res Protoc.

[19] Nittas V, Mütsch M, Ehrler F, Puhan MA (2018). Electronic patient-generated health data to facilitate prevention and health promotion: a scoping review protocol. BMJ Open.

[20] Hensher DA, Rose JM, Greene WH (2005). Applied choice analysis: a primer.

[21] Aizaki H, Nishimura K (2008). Design and Analysis of Choice Experiments Using R: A Brief Introduction. Agricultural Information Research.

[22] The R Project for Statistical Computing. R Foundation.

[23] Walsh S, O'Shea E, Pierse T, Kennelly B, Keogh F, Doherty E (2020). Public preferences for home care services for people with dementia: A discrete choice experiment on personhood. Soc Sci Med.

[24] Johnson R, Orme B (2003). Getting the Most from CBC. Sequim: Sawtooth Software Research Paper Series.

[25] McFadden D (1973). Conditional logit analysis of qualitative behavior.

[26] Rezaei A, Patterson Z (2015). Detecting, NonTransitive, Inconsistent Responses in Discrete Choice Experiments. CIRRELT.

[27] Janssen EM, Hauber AB, Bridges JFP (2018). Conducting a Discrete-Choice Experiment Study Following Recommendations for Good Research Practices: An Application for Eliciting Patient Preferences for Diabetes Treatments. Value Health.

[28] Nittas V, Lun P, Ehrler F, Puhan MA, Mütsch M (2019). Electronic Patient-Generated Health Data to Facilitate Disease Prevention and Health Promotion: Scoping Review. J Med Internet Res.

[29] Kim J (2014). A qualitative analysis of user experiences with a self-tracker for activity, sleep, and diet. Interact J Med Res.

[30] Dunstone K, Conway C (2014). There is an app for that! Communicating UV via the SunSmart app. https://pdfs.semanticscholar.org/bb37/8e7c335ea19ef0aa33d9d4fc7b884da99a9b.pdf.

[31] Rodrigues AM, Sniehotta FF, Birch-Machin MA, Olivier P, Araújo-Soares V (2017). Systematic and Iterative Development of a Smartphone App to Promote Sun-Protection Among Holidaymakers: Design of a Prototype and Results of Usability and Acceptability Testing. JMIR Res Protoc.

[32] Tang J, Abraham C, Stamp E, Greaves C (2015). How can weight-loss app designers' best engage and support users? A qualitative investigation. Br J Health Psychol.

[33] Pfeiffer J, Von Entress-Fürsteneck M, Urbach N, Buchwald A (2016). Quantify-ME: Consumer acceptance of wearable self-tracking devices.

[34] Xie W, Lewis WM, Kaser J, Ross Welch C, Li P, Nelson CA, Kothari V, Terry BS (2017). Design and Validation of a Biosensor Implantation Capsule Robot. J Biomech Eng.

[35] Banerjee S, Hemphill T, Longstreet P (2018). Wearable devices and health care: Data sharing and privacy. The Information Society.

[36] Anderson K, Burford O, Emmerton L (2016). Mobile Health Apps to Facilitate Self-Care: A Qualitative Study of User Experiences. PLoS One.

[37] Struik LL, Bottorff JL, Baskerville NB, Oliffe JL (2018). The Crush the Crave Quit Smoking App and Young Adult Smokers: Qualitative Case Study of Affordances. JMIR Mhealth Uhealth.

[38] Stawarz K, Cox A, Blandford A (2014). Don't forget your pill! Designing effective medication reminder apps that support users' daily routines.

[39] Pich J (2019). Patient reminder and recall interventions to improve immunization rates: A Cochrane review summary. Int J Nurs Stud.

[40] Yuan S, Ma W, Kanthawala S, Peng W (2015). Keep Using My Health Apps: Discover Users' Perception of Health and Fitness Apps with the UTAUT2 Model. Telemed J E Health.

[41] Gowin M, Cheney M, Gwin S, Franklin WT (2015). HealthFitness App Use in College Students: A Qualitative Study. American Journal of Health Education.

[42] Dinsmore JB, Swani K, Dugan RG (2017). To “free” or not to “free”: Trait predictors of mobile app purchasing tendencies. Psychology & Marketing.

